# A pharmacokinetic and pharmacodynamic study on metronomic irinotecan in metastatic colorectal cancer patients

**DOI:** 10.1038/sj.bjc.6604311

**Published:** 2008-03-25

**Authors:** G Allegrini, A Falcone, A Fioravanti, M T Barletta, P Orlandi, F Loupakis, E Cerri, G Masi, A Di Paolo, R S Kerbel, R Danesi, M Del Tacca, G Bocci

**Affiliations:** 1 Division of Medical Oncology, General Hospital of Livorno, Department Of Oncology, University of Pisa, Pisa, Italy; 2 Division of Pharmacology and Chemotherapy, Department of Internal Medicine, University of Pisa, Pisa, Italy; 3 Molecular and Cellular Biology Research, Sunnybrook Health Sciences Centre, University of Toronto, Toronto, Ontario, Canada; 4 Department of Medical Biophysics, University of Toronto, Toronto, Ontario, Canada

**Keywords:** metronomic chemotherapy, angiogenesis, irinotecan, colon cancer, clinical study, thrombospondin-1

## Abstract

The pharmacokinetics (PK) and pharmacodynamics (PD) of metronomic irinotecan have not been studied in cancer patients. The aim of the study is to investigate the PK/PD profile of irinotecan/SN-38 administered by metronomic schedule. Twenty chemotherapy-refractory or chemotherapy-resistant patients with metastatic colorectal carcinoma were enrolled. Irinotecan was infused continuously as follows: irinotecan 1.4 mg m^−2^ day^−1^ (*n*=7), 2.8 mg m^−2^ day^−1^ (*n*=5) and 4.2 mg m^−2^ day^−1^ (*n*=8). Drug levels were examined by HPLC, whereas ELISAs and real-time RT-PCR were used, respectively, for the measurement of plasma levels and gene expression in peripheral blood mononuclear cells of vascular endothelial growth factor/thrombospondin-1. Pharmacokinetic analysis demonstrated that the steady-state levels (*C*_ss_) of SN-38 were between 1 and 3.3 ng ml^−1^. From a PD point of view, higher thrombospondin-1 (TSP-1) plasma levels (153.4±30.1 and 130.4±9.2% at day 49 *vs* pretreatment values at 1.4 and 2.8 mg m^−2^ day^−1^ dose levels, respectively) and increased gene expression in PBMC were found during the metronomic irinotecan infusion, especially at the lower doses. Four patients (20%) obtained a stable disease (median 3.9 months) despite progressing during previous standard irinotecan schedule. Toxicities >grade 1 were not observed. Metronomic irinotecan administration is very well tolerated and induces an increase of gene expression and plasma concentration of TSP-1 at low plasma SN-38 concentrations.

Chemotherapy administration using long-term, continuous and low-dose schedule has been recently introduced in the therapy of solid tumours. Colleoni and co-workers have used the metronomic/antiangiogenic strategy, mainly based on the use of daily oral cyclophosphamide (CTX) in combination with low-dose methotrexate given 2 days/week, in clinical trials for the treatment of metastatic breast cancer patients, and reported promising clinical activity in the absence of serious adverse events (Colleoni *et al*, 2002, 2006; Orlando *et al*, 2006a, 2006b). Moreover, the low-toxicity profile (Kerbel and Kamen, 2004) and the low costs (Bocci *et al*, 2005b) of the metronomic CTX regimens enhanced the quality of life of patients and suggested immediate potential use in various clinical settings. Glode *et al* (2003) and Vogt and co-workers (Vogt *et al*, 2003; Coras *et al*, 2004) studied a metronomic chemotherapy schedule based on alkylating agents (CTX and trofosfamide, respectively) in combination with drugs thought to have some antiangiogenic effects (i.e., dexamethasone, rofecoxib and pioglitazone), demonstrating efficacy as a salvage therapy in the treatment of patients with hormone-refractory prostate carcinoma (Glode *et al*, 2003) or palliative treatment of patients with advanced malignant vascular tumours (Vogt *et al*, 2003) and endemic Kaposi sarcoma (Coras *et al*, 2004). More recently, the metronomic administration of CTX or vinblastine (Stempak *et al*, 2006) was studied in paediatric cancer patients, while temozolomide (Baruchel *et al*, 2006; Stempak *et al*, 2006) was administered in children with recurrent/refractory brain tumours without severe toxicities and with positive results. Continuous oral thalidomide and celecoxib with alternating oral etoposide and CTX have been also studied in paediatric cancer patients (Kieran *et al*, 2005). Garcia *et al* (2008) have recently reported encouraging phase II trial results of metronomic cyclophosphamide, administered daily, in combination with bevacizumab given every 2 weeks, for treatment of recurrent ovarian cancer.

As stated above, preclinical and clinical experiences on metronomic chemotherapy are so far almost mostly focused on low-dose CTX; therefore, studies on other chemotherapeutic drugs are urgently needed. Moreover, rational and less empirical approaches to the clinical development of new metronomic schedules could help setting a more rigorous standard into this evolving and growing field of chemotherapy.

Drugs affecting pathological angiogenesis represent a new and promising approach to metastatic colorectal cancer (mCRC), as shown by the results of the bevacizumab-based phase III clinical studies (Hurwitz *et al*, 2005). Further evaluations of antiangiogenic regimens in preclinical and clinical studies have considerable potential to improve prognosis and quality of life of patients with mCRC. Over the last few years, the introduction of new chemotherapeutic drugs such as irinotecan has resulted in improved prognosis of patients with mCRC (Holen and Saltz, 2001). Despite abundant information about the pharmacology of irinotecan (Di Paolo *et al*, 2006), and its active metabolite SN-38, on cancer cells using different therapeutic approaches, no data are currently available about clinical effects of metronomic irinotecan administration.

Based on this background, we decided to perform a pharmacokinetic/pharmacodynamic (PK/PD) study in 20 patients with mCRC, heavily pretreated with both irinotecan- and oxaliplatin-based chemotherapy, to investigate the (i) PK of metronomic irinotecan/SN-38 at different dose levels; and (ii) changes in antiangiogenic (thrombospondin-1 (TSP-1)) and proangiogenic factors (vascular endothelial growth factor (VEGF)) during treatments.

## MATERIALS AND METHODS

### Patient selection

The study was approved by the local ethics committee and registered in the European Clinical Trial Database EudraCT (http://eudract.emea.europa.eu; EudraCT registration number 2006-001045-34), and patients were informed of the investigational nature of the study and provided their written informed consent. Patients with a confirmed histological diagnosis of mCRC with no operable disease were studied (Table 1). Other main eligibility criteria included (1) previous chemotherapy with fluoropyrimidines, oxaliplatin, irinotecan; (2) measurable disease progressing during (refractory patients) or within 3 months (resistant patients) from the end of the treatments; (3) age ⩽75 years; (4) ECOG performance status of ⩽2; (5) adequate bone marrow, renal and liver function (leukocyte count ⩾3000 mm^−3^, platelet count ⩾100 000 mm^−3^, serum creatinine ⩽1.3 mg dl^−1^, serum bilirubin ⩽1.5 mg dl^−1^, AST and ALT ⩽2.5 times normal values); (6) life expectancy of more than 3 months. Exclusion criteria were as follows: brain metastasis, symptomatic cardiac disease, recent history of myocardial infarction, active infections and inflammatory bowel disease.

### Treatment schedule and doses

Irinotecan was administered as continuous i.v. infusion. Its administration required the implant of a central venous catheter and the use of an external programmable pump (Deltec CADD-Plus, St Paul, MN, USA). Every week external volumetric pumps were refilled with the weekly dose of irinotecan dissolved in NaCl 0.9%. Under these conditions, irinotecan is stable for extended periods of time (Li and Koda, 2002). To define the optimal metronomic dose of irinotecan, we calculated the weekly dose intensity (DI) of irinotecan when administered with the schedule proposed by Herben *et al* (1999) (39.4 mg m^−2^; irinotecan is given 3 out of 4 weeks continuously) that corresponded to a daily dose of irinotecan of 5.6 mg m^−2^; therefore, we chose three different dose levels of irinotecan, starting from a reduction of 75%, followed by the 50 and 25% with respect to the calculated dose of 5.6 mg m^−2^ day^−1^ previously reported. The doses of irinotecan administered by metronomic schedule for each group of patients were as follows:

1. 1.4 mg m^−2^ day^−1^ (DI=9.8 mg m^−2^ week^−1^, number of patients=7)
2. 2.8 mg m^−2^ day^−1^ (DI=19.6 mg m^−2^ week^−1^, number of patients=5)
3. 4.2 mg m^−2^ day^−1^ (DI=29.4 mg m^−2^ week^−1^, number of patients=8)

Neither antiemetic premedication nor prophylactic treatment with granulocyte colony-stimulating factor (G-CSF) was administered. The patients continued the treatment until progression of disease or patient's consent withdrawal.

### Clinical assessment, toxicity and response criteria

Pretreatment evaluation included history and physical examination, performance status assessment, complete blood cell with differential and platelet counts, complete blood profile, tumour markers, urinalysis, ECG, chest X-ray or computed tomography scan, abdominal computed tomography scan and/or sonogram, and any other appropriate diagnostic procedure to evaluate metastatic sites. During treatment, a physical examination, a complete blood cell count, blood profile, urinalysis and toxicity evaluation were performed every week, immediately before pump refilling. Sites of metastatic disease were radiologically re-evaluated every 2 months, according to the RECIST criteria (Therasse *et al*, 2000). A chest X-ray and/or an abdominal sonogram were repeated at least every 6 months if there was no evidence of lung or abdominal disease, respectively. Toxicities were scored according to the standard NCI Common Terminology Criteria for Adverse Events, version 3.0. Duration of responses was calculated from the first day of treatment to the date of first observation of progressive disease or last examination.

### Pharmacokinetics of metronomic irinotecan, SN-38 and SN-38 glucuronide

The PK analysis of irinotecan and its main metabolites SN-38 and SN-38 glucuronide (SN-38glu) was performed as previously described (Masi *et al*, 2004) with minor modifications. Blood samples (4 ml each) for drug assays were taken from an indwelling i.v. cannula placed in an antecubital vein at baseline, 30 min, 1 h, and at days 7, 14, 21, 28, 35, 42, 49, 56 and 63 after the beginning of irinotecan i.v. infusion; at day 63, at the end of the infusion and before refilling the pump, the blood sampling was performed after 30 min, 2 and 6 h. Blood was collected in heparinised tubes (Vacutainer tubes; Becton Dickinson Vacutainer System) and then centrifuged (10 min, 4000 r.p.m., 4°C) to separate plasma, which was stored at −20°C and assayed within 1 week. Briefly, concentration of irinotecan and SN-38 was evaluated after extraction of 1 ml of plasma with methanol containing 0.1% HCl (10 N); the samples were then centrifuged and the clear supernatant was evaporated to dryness under nitrogen flow in a thermostated bath at 45°C. The resulting pellet was reconstituted in methanol acidified with 0.1% HCl (10 N) and eluted through a μBondapack C_18_ stationary phase column (300 × 3.9 mm, 10 μm; Waters, Milford, MA, USA) by KH_2_PO_4_ (0.1 M)/acetonitrile (60 : 40, v/v; pH 6.0) containing sodium heptansulphonate 3 mmol l^−1^. The chromatographic system LC Module I Plus (Waters) was equipped with a Model 474 scanning fluorescence detector with excitation and emission wavelengths set at 375 and 525 nm, respectively. Data analysis was performed using Millennium 2.1 software (Waters). The SN-38glu plasma concentration was measured after incubation of plasma samples with β-glucuronidase (10 UI μl^−1^ of plasma) at 37°C for 2 h before extraction. The difference between peak areas corresponding to SN-38 in β-glucuronidase-treated *vs* untreated samples corresponded to the plasma levels of SN-38glu. Standard calibration curves were generated on each day of analysis by adding irinotecan and SN-38 to 1 ml of blank plasma obtained from healthy donors, resulting in final concentrations that ranged from 12 500 to 0.8 ng ml^−1^ and 2500 to 0.16 ng ml^−1^ for irinotecan and SN-38, respectively. The range of linearity of the HPLC method was from 0.8 to 12 500 ng ml^−1^ for irinotecan and 2500 to 0.16 ng ml^−1^ for SN-38.

Individual plasma concentration profiles of irinotecan and its catabolites were fitted according to a two-compartment open model by means of the WinNonlin 5.1 computer software (Pharsight Corporation, Mountain View, CA, USA) The AUC of irinotecan (CPT-11), SN-38 and SN-38glu from 0 to 63.25 days was calculated by the log-linear trapezoidal method until the last sampling time. Peak plasma concentration (*C*_max_) was obtained by visual inspection of the concentration *vs* time profile of irinotecan and metabolites, whereas steady-state concentrations (*C*_ss_) were calculated as mean values of plasma levels at days 7, 14, 21, 28, 35, 42, 49, 56 and 63 of infusion. Finally, the clearance of irinotecan (CL) was determined as the infusion rate of the drug divided by its *C*_ss_ value. The relative extent of metabolic conversion (REC) of irinotecan was calculated as AUC_SN-38_/AUC_CPT-11_, whereas the drug metabolic ratio (MR) was obtained as (AUC_SN-38_+AUC_SN-38glu_)/AUC_CPT-11_. The glucuronidation ratio (GR) was calculated as the AUC_SN-38glu_/AUC_SN-38_ to obtain an indirect estimate of the activity of glucuronidation of the active metabolite SN-38, and the biliary index (BI) was evaluated as (AUC_CPT-11_) × (AUC_SN-38_/AUC_SN-38glu_).

### Assessment of human *VEGF* and *TSP-1* gene expression and plasma levels

Before drug administration and at days 7, 14, 21, 28, 35, 42, 49, 56 and 63, 10 ml of blood was drawn from the antecubital vein of patients. Peripheral blood mononuclear cells (PBMC) were collected as described (Bocci *et al*, 2006) and the cell suspension was centrifuged at 1000 r.p.m. for 10 min and washed with PBS; the resulting pellet was immediately frozen in liquid nitrogen and stored at −80°C. Briefly, RNA (1 μg) was reverse transcribed (Bocci *et al*, 2005a) and the resulting cDNA was diluted (2 : 3) and then amplified by QRT-PCR with the Applied Biosystems 7900HT sequence detection system. Vascular endothelial growth factor- and TSP-1-validated primers were purchased from Applied Biosystems (Assay ID Hs00170236_m1 and Hs00173626_m1, respectively). The PCR thermal cycling conditions and optimisation of primer concentrations were followed as *per* the manufacturer's instructions. Amplifications were normalised to GAPDH and the quantitation of gene expression was performed using the ΔΔ*C*_t_ calculation; the amounts of VEGF and TSP-1, normalised to the endogenous control and relative to the calibrator (PBMC sample at day 0), are given as 2^−ΔΔ*C*_t_^. The data are presented as the percentage of 2^−ΔΔ*C*_t_^ before the beginning of irinotecan infusion.

Plasma samples obtained at the same days of PBMC collection were assessed for TSP-1 and VEGF levels using the commercially available ELISA and EIA kits. Each sample was assayed for human VEGF and TSP-1 concentrations by the ELISA Kit Quantikine® (R&D Systems, USA) and by the ChemiKine™ Human TSP-1 EIA Kit (Chemicon, Temecula, CA, USA), respectively. Measurements were performed by the microplate reader Multiskan Spectrum (Thermo Labsystems, Milan, Italy) set to 450 nm (with a wavelength correction set to 540 nm) for the VEGF kit and 490 nm for TSP-1 kit. The data are presented as the percentage of the VEGF and TSP-1 plasma levels at day 0.

### Statistical analysis

Since the study was exploratory in nature, no statistical hypothesis testing has been performed. Moreover, the sample size (20 patients) has been judged to be adequate based on the suggestions of the entropy-based approach to sample size in translational clinical trials (Piantadosi, 2005). The analysis by ANOVA, followed by the Student–Newman–Keuls test, was used to assess the statistical differences of data. *P*-values lower than 0.05 were considered significant. Statistical analyses were performed using the GraphPad Prism software package version 4.0 (GraphPad Software Inc., San Diego, CA, USA).

## RESULTS

### Patients and toxicity

As outlined in Table 1, 20 patients with advanced mCRC entered the study. Median age was 71 years (range, 51–79 years), ECOG performance status was 0–1 in 18 patients and 2 in one. As reported, the entire study population was heavily pretreated and irinotecan refractory or resistant. Of note, 35% of patients had also received a cetuximab-based therapy. Overall, 188 weeks of irinotecan were administered by metronomic schedule with a median of 9 weeks per patient (range, 2–20 weeks). The end of treatment was due to disease progression in all patients.

All patients were assessable for toxicities. Toxicities were very uncommon. In particular, we did not observe toxicities higher than grade 1. Three (15%) and five patients (25%) experienced, respectively, a transient grade 1 diarrhoea and nausea, resolved without interrupting the treatment. No haematological toxicities were observed.

### Antitumour activity and survival

**Figure 1:**
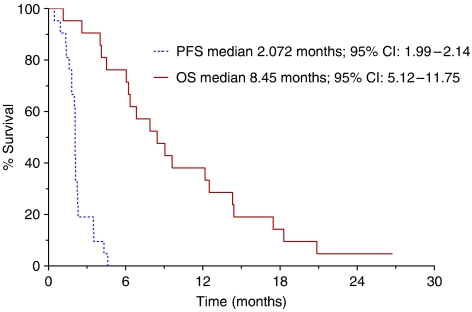
Actuarial PFS and OS curves calculated by the Kaplan–Meier method from the first day of metronomic irinotecan chemotherapy.

All 20 patients were assessable for response to treatment. Four patients treated at 1.4 mg m^−2^ day^−1^ (1 patient), 2.4 mg m^−2^ day^−1^ (2 patients) and 4.2 mg m^−2^ day^−1^ (1 patient) obtained a stabilisation of disease that lasted a median period of 3.9 months (range, 3–5 months). In the remaining 16 patients, disease progression was observed at the first clinical evaluation. After a median follow-up of 20 months, median progression-free survival (PFS) was 2.07 months (95% CI: 1.99–2.14) and median overall survival (OS) was 8.4 months (95% CI: 5.1–11.7); curves estimated by the Kaplan–Meier method from the first day of treatment are reported in Figure 1.

### Pharmacokinetics of metronomic irinotecan, SN-38 and SN-38glu

**Figure 2:**
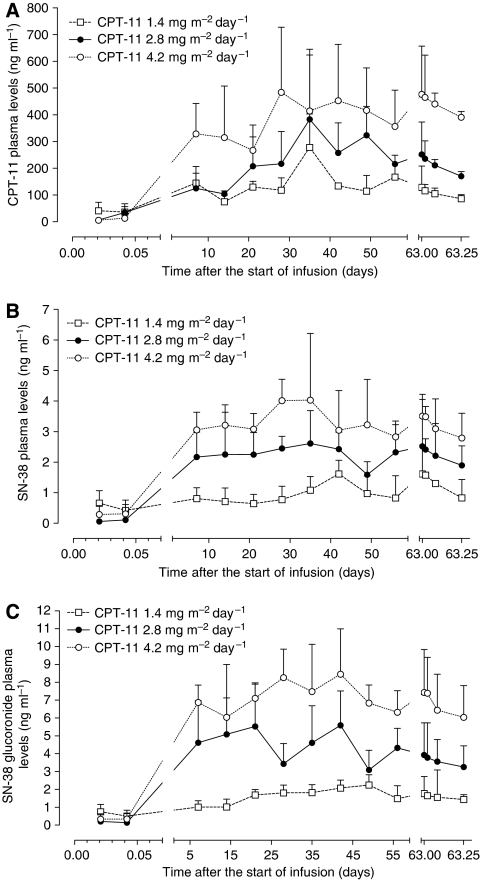
Plasma levels of irinotecan (CPT-11) (**A**), SN-38 (**B**) and SN-38 glucuronide (**C**) in 20 mCRC patients receiving an i.v. continuous infusion of CPT-11 at three different dose levels. Symbols and bars represent mean and s.d.

Main PK parameters of irinotecan and its metabolites are reported in Table 2, whereas the plasma profiles of irinotecan, SN-38 and SN-38glu at the different infusion schedules are shown in Figure 2A–C, respectively. Pharmacokinetic analysis demonstrated that the *C*_ss_ of irinotecan 1.4, 2.8 and 4.2 mg m^−2^ day^−1^ were 143.1±56.8, 231.6±101.4 and 390±171 ng ml^−1^, respectively, whereas those of SN-38 were 1.00±0.52, 2.29±0.87 and 3.33±0.96 ng ml^−1^, respectively, and resulted statistically different among them (*P*<0.05). Moreover, the *C*_ss_ of SN-38glu were, as expected, higher than the ones of SN-38. Pharmacokinetic analysis of irinotecan showed an increased metabolism of the parent drug into the active metabolite SN-38 when higher doses were administered (Figure 2B). As expected, irinotecan AUC value was higher at the 4.2 dose with respect to the 2.8 and 1.4 doses, even though it was not statistically significant, whereas mean AUC value of SN-38 was significantly lower at 1.4 dose than at 2.8 and 4.2 doses (*P*<0.05) (Table 2). The comparison of PK of irinotecan at different doses did not reveal any significant difference among *C*_max_ and *t*_1/2_β values (Table 2). Instead, significant differences were found for *C*_max_ values of SN-38 and SN-38glu at different irinotecan doses. Further analysis demonstrated that the higher doses led to an increase in BI, REC and MR values, even if only the increase of BI was statistically significant (*P*<0.05). Instead, GR value did not significantly increase after administration of higher doses of irinotecan (Table 2).

### Changes in TSP-1 and VEGF plasma and gene expression in PBMC

**Figure 3:**
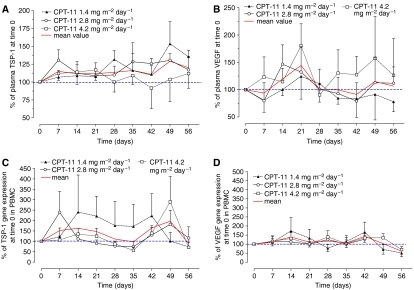
TSP-1 (**A**) and VEGF (**B**) plasma concentrations and TSP-1 (**C**) and VEGF (**D**) gene expression in PBMC of patients administered with three different metronomic irinotecan (CPT-11) doses. Symbols and bars represent mean and s.d. The data are presented as a percentage of the concentration at day 0 (before the beginning of CPT-11 infusion) of each individual patient or as the percentage of 2^−ΔΔ*C*_t_^ at day 0 of each single patient.

To compare the variations of plasma TSP-1 and VEGF before and during the metronomic treatments, graphs were drawn to show the concentrations as a percentage of the concentration at day 0 of individual patients before the starting of irinotecan infusion. Figure 3A shows that TSP-1 levels markedly increased in treated patients and remain consistently higher for more than 8 weeks. However, differences were noted for TSP-1 concentrations among the three dose levels: the average TSP-1 increase was higher at the lower doses of 1.4 and 2.8 mg m^−2^ day^−1^ (Figure 3A) reaching maximum increments at day 49 of 153.4±30.9 and 130.5±9.3%, respectively, *vs* 100% of day 0. In contrast, TSP-1 concentrations in patients treated with CPT-11 at 4.2 mg m^−2^ day^−1^ returned to the baseline values after an initial increase (Figure 3A). Figure 3B shows the results of the different profiles of VEGF plasma levels in the treated patients. Mean plasma VEGF levels, although with a high variability, increased in the first 3 weeks of treatment, whereas after day 28 they returned to baseline levels (Figure 3B). However, differences were noticed among the three dose levels: at the lowest dose (1.4 mg m^−2^ day^−1^) VEGF concentrations decreased (at day 56, 77.9±18 *vs* 100% of day 0), whereas at the highest dose (4.2 mg m^−2^ day^−1^) they increased (at day 56, 126.3±67 *vs* 100% of day 0) during the CPT-11 infusion. Interestingly, only at lower metronomic doses, there was a simultaneous increase in TSP-1 levels and a decrease of VEGF concentrations, which would suggest a shift to an antiangiogenic state.

Figure 3C and D shows TSP-1 and VEGF gene expression profiles in PBMC, a normal cell compartment. Differences were found in TSP-1 gene expression among the three dose levels. Indeed, the irinotecan 1.4 mg m^−2^ day^−1^ determined a marked increase in TSP-1 gene expression in PBMC at least until day 42 (222.4±106.9 *vs* 100% of day 0), whereas at the other doses a marked increase was seen only after day 35 (Figure 3C). In contrast, VEGF expression profile in PBMC was similar to baseline, with the exception of an initial increase (at day 14, 172.6±73.3 *vs* 100% of day 0 for 1.4 mg m^−2^ day^−1^ dose).

## DISCUSSION

The present study described, for the first time, the PK of metronomic irinotecan and demonstrated a marked increase in TSP-1 plasma concentrations and gene expression (already at lower irinotecan dose levels), suggesting a possible use of this PD marker in irinotecan-based metronomic treatment strategies. Moreover, low-dose metronomic irinotecan was not toxic and potentially active in heavily pretreated, refractory or resistant population of mCRC patients.

Based on promising antiangiogenic and antitumour preclinical results (Bocci *et al*, 2007), we decided to test the metronomic irinotecan schedule in the clinic, specifically in patients with mCRC resistant to irinotecan standard doses. The ‘ethical condition’ to evaluate the clinical effect of an irinotecan metronomic treatment in this patient population depended on the fact that there was no evidence that a third/fourth line of chemotherapy could produce an improvement in terms of clinical benefit or efficacy in patients with mCRC already treated with both oxaliplatin- and irinotecan-based chemotherapy. The results of several phase II clinical studies showed that a third/fourth line of fluoropyrimidine-based chemotherapy in this setting of patients generally produces a poor response rate (around or less than 10%), with a median PFS between 2 and 3 months and with a median OS of approximately 6–9 months (Chong *et al*, 2005; McCollum *et al*, 2006; Scartozzi *et al*, 2006). Besides the weak antitumour activity, these treatments are limited by a substantial toxicity ⩾grade 3 (NCI scale) reported in about 10–15% of all patients. The role of the targeted therapy in these setting of patients has also been evaluated. Chen *et al* (2006) have recently published the results of a large multicentre trial of bevacizumab in combination with 5-fluorouracil/leucovorin in patients with mCRC pretreated with both oxaliplatin- and irinotecan-based chemotherapy. The results have shown a response rate of 4%, a median PFS of 3.5 months and a median OS of 9 months. Of note, adverse events ⩾grade 3 were reported in 47% of all treated patients.

More promising results seem to come from the use of the monoclonal antibodies against the epidermal growth factor receptor cetuximab and panitumumab in this setting of patients. Jonker *et al* (2007) have evaluated, in a phase III clinical study, the role of cetuximab in patients with mCRC pretreated with an oxaliplatin- and irinotecan-based chemotherapy, showing an improvement in terms of OS of cetuximab plus best supportive care (BSC) respect to BSC alone (median OS of 6.1 *vs* 4.6 months, *P*=0.0046). Van Cutsem *et al* (2007) in a similar setting of patients have compared panitumumab plus BSC to BSC alone, showing that panitumumab prolonged the PFS (HR: 0.54; 95% CI: 0.44–0.66, *P*<0.0001), with no difference in terms of OS. Despite the promising results, some issues could limit the use of these therapies in this setting of patients, such as the low impact on survival, the high cost of the drugs and the significant higher rate of grade 3–4 adverse events with respect to BSC. Thus, the study of metronomic chemotherapy could be of interest in heavily pretreated mCRC. A recent study has evaluated the role of a combination metronomic treatment with CTX, vinblastine and rofecoxib in patients with advanced tumours, in which 13 patients with diagnosis of mCRC were included. One patient had a partial response, while another patient had a stable disease; the time to progression of these patients was 12 and 7 months, respectively (Young *et al*, 2006). These results suggest a potential antitumour effect of the metronomic approach in patients with mCRC.

One of the major concerns regarding the clinical application of metronomic chemotherapy relates to the dosing levels and the frequency of administration of the chemotherapeutic drugs. This important issue has generated confusion regarding the term ‘metronomic’ that has been sometimes associated with a simple, small reduction of a standard dose. Moreover, among the published trials, the dose for the metronomic chemotherapy is often arbitrarily chosen (e.g., the ‘classic’ fixed dose of 50 mg day^−1^ of CTX simply corresponds to a single tablet of the commercially available medicament) and no further efforts have been made to define the best dose. In our study, we evaluated three different dose levels of metronomic irinotecan that was infused continuously without breaks, starting from a reduction of 75% of the maximum tolerable dose of irinotecan when infused continuously more than 21 days every 28 days reported by Herben *et al* (1999). Moreover, we thoroughly investigated the changes in TSP-1 and VEGF expression/secretion in accessible compartments in metastatic patients such as peripheral mononuclear cells and plasma to confirm our preclinical data and find indexes of biological activity of the CPT-11 metronomic treatment.

Although our study was not comparative, the results show that the irinotecan metronomic chemotherapy produces clinical results such as those observed with other schedules of third/fourth line of treatment in patients with a diagnosis of metastatic colorectal carcinoma, both in terms of median PFS and median OS. Furthermore, our results were accompanied by a total absence of toxicity and in a heavily pretreated, irinotecan-resistant population with progressive disease. Moreover, G-CSF was not administered to patients receiving metronomic irinotecan chemotherapy and as such this could have an advantage, not only in terms of cost but also in terms of avoiding the possibility that the exogenous G-CSF might promote angiogenesis by mobilising circulating endothelial progenitor cells (CEPs) (Natori *et al*, 2002; Shaked and Kerbel, 2007). Our results suggest that metronomic irinotecan chemotherapy could work through a mechanism of action that is not related to the direct cytotoxic effect on tumour cells but rather through an antiangiogenic activity targeting proliferating endothelial cells as shown in the preclinical setting. Indeed, higher TSP-1 plasma levels were found during the metronomic irinotecan infusion when compared to the baseline values in single patients as well as the increased gene expression in the PBMC compartment, especially at the lower irinotecan doses. These findings clinically confirmed that low-dose CPT-11 inhibits angiogenesis, in part, by upregulating TSP-1 in tumour endothelial (Bocci *et al*, 2003), or tumour and tumour-associated stromal cells (Hamano *et al*, 2004), promoting endothelial cell apoptosis (de Castro Junior *et al*, 2006) and suppressing the mobilisation of circulating endothelial progenitors (Shaked *et al*, 2005). This linkage between PD parameters and dose levels could open a promising area of clinical investigation on surrogate markers for the activity of the metronomic chemotherapy approach. Indeed, a previous attempt to monitor putative surrogate markers such as VEGF, endostatin and TSP-1 plasma levels during metronomic CTX revealed a high degree of variability and no statistically significant relationships between these markers and disease progression or maintenance of stable disease in paediatric patients (Stempak *et al*, 2006). However, the heterogeneous patient population, the drugs that were involved and the lack of a preclinical investigation of the doses that were used, and combination of drugs used all could have affected the outcome of the results. Colleoni *et al* (2002) described a reduction in serum VEGF of both responders and non-responders in breast cancer metastatic patients treated with metronomic CTX. In our study, the individual plasma VEGF concentrations of patients initially increased when compared to the baseline values in the first 3 weeks and then at lower doses constantly decreased. Despite the presence of a high variability, VEGF plasma levels may reflect both the initial elevated hypoxia of tumour tissue caused by the antiangiogenic therapy, as previously shown (Bocci *et al*, 2004; Motzer *et al*, 2006), and the low rate of VEGF secretion by tumour or tumour-associated stromal cells due to the long-term therapy (Colleoni *et al*, 2002).

This PK/PD study underlines the importance to conduct further comparative studies with BSC or bevacizumab to establish the feasibility of metronomic irinotecan approach. Moreover, this suggests further clinical steps that might be explored such as the administration of rubitecan, an oral camptothecin (Clark, 2006), or combination studies with other low-dose oral chemotherapeutic drugs already approved for colorectal cancer such as UFT (Munoz *et al*, 2006) or capecitabine. In addition, combination of a targeted antiangiogenic drug such as bevacizumab with metronomic irinotecan therapy might also be considered in patients not previously treated with this drug since such combinations show much greater antitumour efficacy compared to metronomic chemotherapy alone or the antiangiogenic drug alone (Klement *et al*, 2000; Kerbel and Kamen, 2004; Pietras and Hanahan, 2005).

## Figures and Tables

**Table 1:** Patient characteristics

*n*	20
Median age (years) (range)	71 (51–79)
Gender (male/female)	11/9
ECOG (0/1/2)	8/11/1
	
*Primary tumour site (n)*	
Colon	16
Rectum	4
	
*Metastatic sites (n)*	
Liver	16
Lungs	13
Peritoneum	4
Lymph nodes	3
Others	3
	
*No. of metastatic sites* *(n)*	
Single	7
Multiple	13
	
*Previous chemotherapy (%)*	
Irinotecan based	100
Fluoropyrimidine based	100
Oxaliplatin based	100
Cetuximab+irinotecan	35
	
*No. of previous cancer treatments for advanced disease (%)*	
Two	55
More than two	45
	
*Irinotecan dose (mg m* ^*−2*^ * day* ^*−1*^ *) (n)*	
1.4	7
2.8	5
4.2	8
	
Median CEA (ng ml^−1^) (range)	62.3 (0.8–5104)
Median body surface area (m^2^) (range)	1.77 (1.64–2)
TSP-1 pretreatment levels (ng ml^−1^) (mean±s.d.)	213.9±110.08
VEGF pretreatment levels (pg ml^−1^) (mean±s.d.)	158.6±83.5

CEA, carcino-embryonic antigen; ECOG, eastern cooperative oncology group; *n*=no. of patients; TSP-1=thrombospondin-1; VEGF=vascular endothelial growth factor.

**Table 2:** Pharmacokinetic parameters of irinotecan, SN-38 and SN-38glu at the doses of irinotecan 1.4, 2.8 and 4.2 mg m^−2^ day^−1^ in 20 patients

	**Mean±s.d.**		
	**1.4 mg m^−2^ day^−1^ (*n*=7)**	**2.8 mg m^−2^ day^−1^ (*n*=5)**	**4.2 mg m^−2^ day^−1^ (*n*=8)**
*Irinotecan*			
AUC (day ng ml^−1^)	8714.7±1564.3	13877.7±3035.2	23051.6±5002.3
CL (ml day^−1^ m^−2^)	154.32±28.4	170.31±44.2	146.11±25.3
*t*_1/2_β (h)	15.9±5.1	20.2±6.2	14.6±3.2
*C*_max_ (ng ml^−1^)	277.6±125.3	382.9±261.8	484.1±243.1
*C*_ss_ (ng ml^−1^)	143.1±56.8	231.6±101.4	390.0±171.0a,b
*T*_max_ (day)	35	35	28
			
*SN-38*			
AUC (day ng ml^−1^)	59.43±7.47	136.21±10.61^c^	200.48±12.26a,b
*t*_1/2_β (h)	18.9±4.3	22.8±6.7	19.9±7.2
*C*_max_ (ng ml^−1^)	1.62±0.45	2.61±1.07	4.03±2.19^a^
*C*_ss_ (ng ml^−1^)	1.00±0.52	2.29±0.87^c^	3.33±0.96a,b
*T*_max_ (day)	42	35	35
			
*SN-38glu*			
AUC (day ng ml^−1^)	100.94±8.82	268.86±14.52^c^	430.10±24.34a,b
*t*_1/2_β (h)	22.31±5.1	17.4±5.6	21.33±6.8
*C*_max_ (ng ml^−1^)	2.24±0.58	5.59±1.91^c^	8.45±2.54a,b
*C*_ss_ (ng ml^−1^)	1.63±0.53	4.42±1.98^c^	7.20±1.59a,b
*T*_max_ (day)	49	42	42
			
BI	5130.1±745	7030.7±537^c^	10744.9±892a,b
REC	0.0068±0.0048	0.0098±0.0035	0.0087±0.0025
MR	0.0184±0.0104	0.0292±0.0183	0.0274±0.0116
GR	1.698±0.206	1.974±0.402	2.145±0.623

AUC=area under the time/concentration curve; BI=biliary index; *C*_max_=maximal plasma concentration; *C*_ss_=steady-state concentration; GR=glucuronidation ratio; MR=metabolic ratio; REC=relative extent of conversion; SN-38glu=SN-38 glucuronide; *t*_1/2_β=terminal half-life; *T*_max_=time to peak.

a *P*<0.05 4.2 *vs* 1.4.

b *P*<0.05 4.2 *vs* 2.8.

c *P*<0.05 2.8 *vs* 1.4.
